# Long-term follow up of opicapone as add-on to levodopa in Parkinson's patients without motor fluctuations

**DOI:** 10.1177/1877718X261436957

**Published:** 2026-04-17

**Authors:** Joaquim J. Ferreira, Olivier Rascol, Fabrizio Stocchi, Angelo Antonini, Joana Moreira, Miguel M. Fonseca, José-Francisco Rocha, Helena C. Brigas, Joerg Holenz, Werner Poewe, on behalf of the Epsilon Study investigators

**Affiliations:** 1 Laboratory of Clinical Pharmacology and Therapeutics, Faculdade de Medicina, Universidade Lisboa, Lisbon, Portugal; 2 CNS – Campus Neurológico, Torres Vedras, Portugal; 3 Clinical Investigation Centre CIC1436 Departments of Neurosciences and Clinical Pharmacology, Centre of Excellence for Neurodegeneration COEN NeuroToul, and NS-Park/FCRIN network; University of Toulouse 3, University Hospital of Toulouse, INSERM, Toulouse, France; 4 Department of Neurology, Institute for Research and Medical Care IRCCS San Raffaele, Roma, Italy; 5 Department of Neurology, San Raffaele University, Rome, Italy; 6 Neurodegenerative Disease Unit, Centre for Rare Neurological Diseases (ERN-RND), Department of Neuroscience, University of Padova, Padova, Italy; 7 Department of Neurology, IRCCS San Camillo, Venice, Italy; 8 BIAL – Portela & Ca S.A., Coronado, Portugal; 9 BIAL - R&D INVESTMENTS, S.A, Porto, Portugal; 10 Department of Neurology, Medical University of Innsbruck, Innsbruck, Austria

**Keywords:** clinical trial, COMT, levodopa, opicapone, Parkinson's disease

## Abstract

Primary results from the 24-week EPSILON study (NCT04978597) showed that adding opicapone to levodopa in non-fluctuating Parkinson's patients significantly improved motor impairment without increasing the development of motor complications versus placebo. All participants finishing the double-blind phase became eligible for open-label treatment with opicapone. Symptomatic efficacy of opicapone was maintained with 1-year open-label treatment (adjusted-mean ± SE change in MDS-UPDRS motor score from double-blind baseline to Week 76: −7.4 ± 0.81); participants who switched from placebo to opicapone had a motor improvement of −6.1 ± 0.79. At Week 76, 80.2% of opicapone-opicapone-treated participants remained motor complication-free versus 69.7% in the placebo-opicapone group (p = 0.1).

## Plain Language Summary

Opicapone as add-on to levodopa in Parkinson's patients without motor fluctuations

**Why was this study done?:**

Levodopa is the most effective drug to improve the motor symptoms of Parkinson's disease (PD), but most patients experience periodic worsening of their symptoms between doses – a phenomenon known as ‘wearing-off’. COMT inhibitors, like opicapone are drugs that prolong the effects of a dose of levodopa and improve-wearing-off fluctuations, but there has been debate about whether they are also beneficial in people who don’t yet have these wearing-off problems but have motor signs or symptoms of PD that require enhanced control.

**>What was studied?:**

Study investigators wanted to see if adding opicapone to levodopa treatment in people with early PD who didn’t yet have motor fluctuations could improve symptoms without increasing the development of motor complications (like as dyskinesia and wearing-off effects). The main study was only 24 weeks, but there was an open-label phase (where participants knew they were taking opicapone) which extended the treatment period for up to 76 weeks.

**What did the study find?:**

In people with early PD, adding opicapone to the levodopa regimen improved motor function and this benefit was sustained over 76 weeks. Most people taking opicapone did not develop motor complications, even after more than a year of treatment. People who switched from placebo to opicapone also improved once they began taking it. Treatment with opicapone was generally safe and well tolerated, with no serious side effects linked to the treatment.

**What does this mean?:**

Adding OPC early in treatment may help people with PD benefit from a longer-lasting motor improvement, without increasing the risk of aggravating or inducing motor complications. It could be a useful option for improving how well levodopa works in people who are still in the early stages of the disease.

Sirs,

We recently reported that the addition of adjunct opicapone (OPC) in non-fluctuating levodopa-treated patients with Parkinson's disease (PD) significantly improved motor impairment without increasing the development of motor complications over 24 weeks versus placebo.^1^ Here we report the findings of the open-label extension phase, which demonstrate the symptomatic effects reported in the double-blind phase^1^ are sustained over at least 76 weeks exposure with >20% improvement in MDS-UPDRS motor scores. Importantly, these sustained symptomatic improvements occurred with only marginal adjustments to levodopa dosing and most participants remained free from motor complications over the extended follow-up period. By week 76, less than 20% of participants who had been treated with OPC for 76 weeks had developed dyskinesias, OFF episodes, and/or dystonia. Participants who switched from placebo to OPC showed similar motor improvement to that observed for OPC participants in the double-blind phase – reenforcing the symptomatic benefits of adding OPC in patients without motor complications. Treatment with OPC continued to be well tolerated.^2^

The Epsilon study^1^ enrolled levodopa-treated PD patients (300–500 mg, 3–4 times daily) diagnosed within 5 years; those with prior motor complications (MDS-UPDRS Part IV score >0) were excluded. In the double-blind phase, participants received placebo or OPC 50 mg as add-on to levodopa for 24 weeks. Those completing this phase entered an open-label extension with OPC 50 mg for up to 52 additional weeks; prior allocation remained blinded until study end.

Of the 322 participants who completed double-blind treatment, 307 (prior-OPC = 152, prior-placebo = 155) entered open-label treatment with opicapone, and 246 (prior-OPC = 120, prior-placebo = 126) completed the study. The most common reasons for study withdrawal were study termination in Ukraine (n = 21) and withdrawal of consent (n = 12). As previously reported,^1^ groups were similar at baseline: mean age 64 years, 63–67% male, disease duration ∼3 years, median Hoehn & Yahr stage 2. Participants were symptomatic with a mean ± SD baseline MDS-UPDRS motor score of 32.9 ± 10.9 for the OPC-OPC group and 33.9 ± 11.2 for the PLC-OPC group. Most participants (56% of those assigned OPC and 65% of those assigned placebo) entered the double-blind phase on levodopa plus another PD therapy; no medication adjustments were allowed until the open-label phase. The early addition of OPC to the levodopa regimen significantly reduced motor symptom severity versus placebo without major impact on levodopa dosing.^1^ At open-label baseline, MDS-UPDRS motor scores were 26.8 ± 12.49 for the OPC-OPC group and 29.8 ± 13.79 for the placebo-OPC group. Mean ± SD levodopa doses were 387 ± 109 mg/day (LEDD of 737 ± 236 mg/day, including OPC) in the OPC-OPC group and 383 ± 89 mg/day (LEDD of 513 ± 195 mg/day) in the placebo-OPC group.

**Figure 1.**
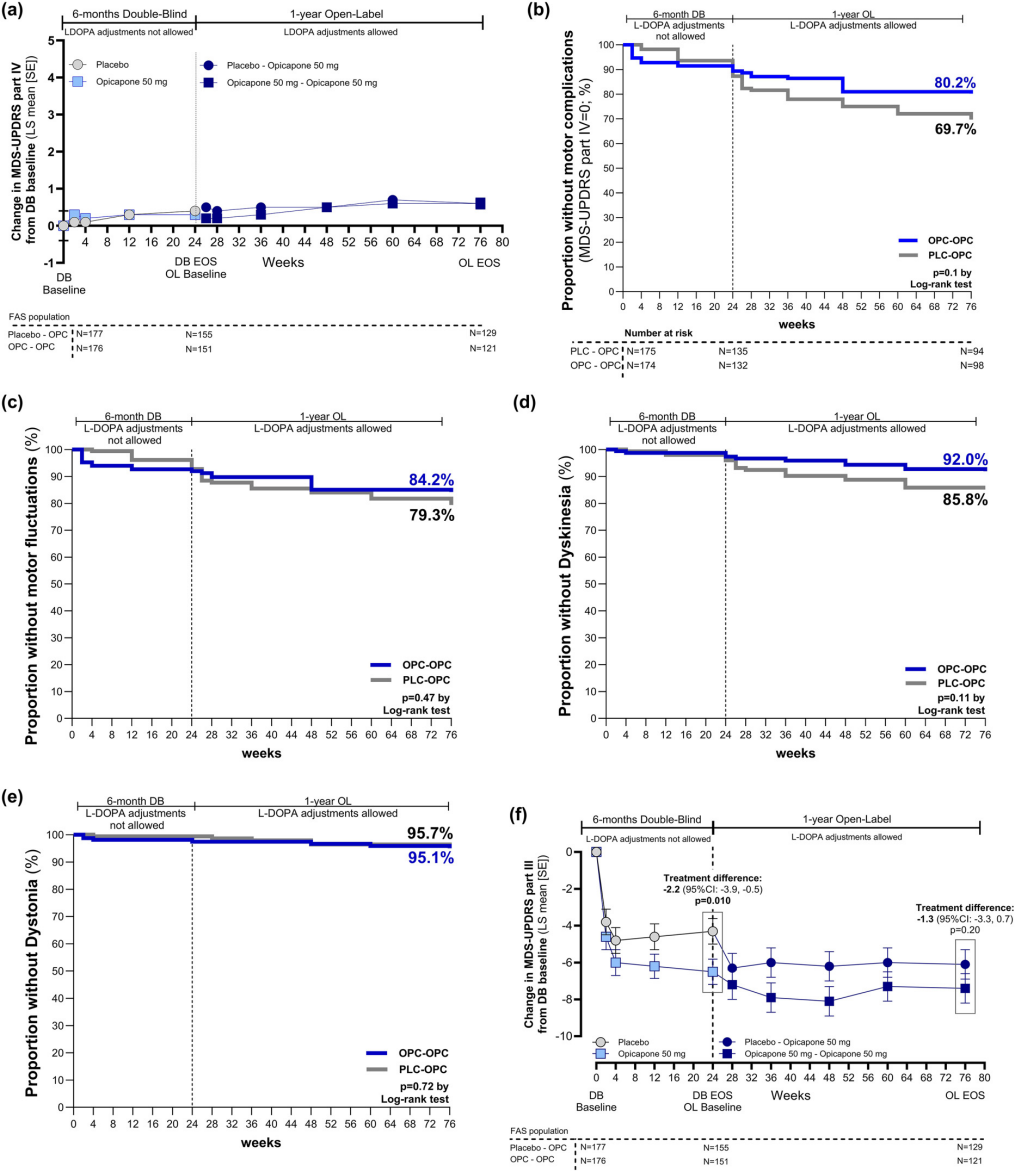
Motor complications: (a) MDS-UPDRS part IV score over time. Kaplan-Meier survival curves for participants remaining free of (b) motor complications (c) OFF episodes (d) dyskinesia, and (e) dystonia; and (f) Change from baseline in MDS-UPDRS part III scores. Legend: *Data in 1a and 1f were evaluated using an MMRM approach with fixed effects for baseline, region, randomized treatment, visit, randomized treatment by visit interaction and baseline by visit interaction, and participant as a random effect*.

In contrast to the STRIDE-PD study with levodopa/carbidopa/entacapone,^3^ earlier and longer opicapone exposure did not significantly increase motor complications. As shown in Figure 1(a), MDS-UPDRS part IV scores for the overall cohort remained low throughout open-label treatment. At study end, scores had increased by 0.6 [95%CI: 0.3, 0.8] points in the OPC-OPC group and 0.6 [95%CI: 0.4, 0.9] points in the placebo-OPC group. Comparing the OPC-OPC group with the placebo-OPC group, the adjusted mean treatment difference (TD) in MDS-UPDRS part IV scores at study end was −0.1 [95%CI: −0.4, 0.3] (*p* = 0.721). Kaplan-Meier survival analysis further showed that a non-significantly greater proportion of participants in the OPC-OPC group remained motor complication-free (MDS-UPDRS part IV score = 0) compared to the placebo-OPC group (80.2% vs 69.7%, log-rank test *p* = 0.1; Figure 1(b)). Similarly, there were no significant differences in the percentage of OPC-OPC or placebo-OPC treated participants remaining free from fluctuations (MDS-UPDRS item 4.3: 84.2% vs. 79.3%, respectively; p = 0.47), dyskinesia (MDS-UPDRS item 4.1: 92% vs. 85.8%, respectively; p = 0.11), or dystonia (MDS-UPDRS item 4.6: 95.1% vs. 95.7%, respectively; p = 0.72) at study end (Figure 1(c)-(e)).

For participants in the OPC-OPC group, the motor improvements on MDS-UPDRS Part III scores reported in the prior double-blind phase^1^ were sustained across the 76 weeks of opicapone treatment (mean ± SE change from open-label baseline of −1.5 ± 0.67 and from double-blind baseline of −7.4 ± 0.81). Participants in the placebo-OPC group showed a consistently smaller reduction of MDS-UPDRS motor scores at each visit compared to those in the OPC-OPC group (Figure 1(f)). Participants who switched from placebo to OPC demonstrated an adjusted mean ± SE improvement of −1.7 ± 0.67 from open-label baseline to week 76 (consistent with OPC treatment effects in the double-blind period) and an overall adjusted mean ± SE change of −6.1 ± 0.79 from double-blind baseline. However, at Week 76, the adjusted mean between group TD of −1.3 [95%CI: −3.3, 0.7] MDS-UPDRS part III points versus double-blind baseline was not significant (p = 0.20).

Of note, the sustained improvements in motor scores with OPC were achieved with little change in levodopa dosage. Regardless of prior treatment allocation, the mean levodopa dose at Week 76 remained approximately 400 mg. By the end of the open-label phase, mean daily levodopa doses had increased by only 35 mg (LEDD +22 mg/day) in the OPC-OPC group and by 27 mg (LEDD +219 mg/day, including adjunct opicapone) in the placebo-OPC group. These findings indicate that optimizing the levodopa regimen with OPC allowed participants to maintain stable treatment without requiring further adjustments to dopaminergic medication or experiencing motor complications. Several studies have demonstrated a strong association between higher levodopa doses and the risk of motor complications, and it is generally recommended to avoid high doses (e.g., >400 mg/day^4^) when not clinically necessary.

Scores for the Non-Motor Symptoms Scale (change from DB baseline OPC-OPC vs placebo-OPC: −0.9 ± 1.2 vs 1.4 ± 1.1), Parkinson's Disease Sleep Scale-2 (−0.4 ± 0.7 vs 0.8 ± 0.6), and the Parkinson's Disease Questionnaire-39 (−0.4 ± 0.7 vs 1.4 ± 0.7) remained stable over 76 weeks of follow-up. Overall, 71.9% of OPC-OPC participants experienced improvements on PGI-I (vs 63.3% in the placebo-OPC group) and 77.7% experienced improvements on CGI-I (vs 68.0%).

Adverse events during open-label treatment were more common in the participants who had switched from placebo to OPC 50 mg than those who initiated OPC earlier (63.9% vs 53.0%, respectively) (Table 1). This is consistent with findings from studies in patients with motor fluctuations,^2^ which show that adverse events typically emerge within the first few weeks of starting OPC and tend to decline thereafter. Participants in the OPC-OPC group were already accustomed to the medication by the time the open-label phase began. The most common TEAEs (placebo-OPC vs OPC-OPC) were ON-OFF phenomenon (n = 15 [9.7%] vs. n = 13 [8.6%]) and dyskinesia (n = 14 [9.0%] vs n = 7 [4.6%]). No treatment-related serious TEAEs were reported. Five participants in the OPC-OPC group died during open-label treatment due to AEs considered unrelated to study treatment (2 for unknown reasons, 1 natural cause, 1 acute respiratory distress syndrome, 1 cardiac arrest).

**Table 1.** Adverse events during open-label treatment with opicapone.

	OPC-OPC, n = 151	PLC-OPC, n = 155
Any TEAE, n (%)	80 (53.0)	99 (63.9)
Treatment-related TEAE, n (%)	16 (10.6)	35 (22.6)
Mild	13 (8.6)	27 (17.4)
Moderate	3 (2.0)	7 (4.5)
Severe	0	1 (0.6)
Serious TEAE, n (%)	17 (11.3)	13 (8.4)
Treatment-related serious TEAE, n (%)	0	0
TEAE leading to death, n (%)	5 (3.3)	0
**Most common (>2%) TEAEs, n (%)**		
ON-OFF phenomenon	13 (8.6)	15 (9.7)
Dyskinesia	7 (4.6)	14 (9.0)
Headache	7 (4.6)	7 (4.5)
Parkinson's disease	6 (4.0)	5 (3.2)
Back pain	1 (0.7)	9 (5.8)
Insomnia	5 (3.3)	5 (3.2)
COVID-19	5 (3.3)	5 (3.2)
Fall	6 (4.0)	2 (1.3)
Arthralgia	5 (3.3)	2 (1.3)
Dizziness	3 (2.0)	2 (1.3)
Urinary tract infection	3 (2.0)	2 (1.3)
Fatigue	4 (2.6)	1 (0.6)
Hypertension	5 (3.3)	0
**TEAE leading to withdrawal, n (%)**	**6 (4.0)**	**4 (2.6)**
Death	3 (2.0)	0
Clostridial sepsis^a^	1 (0.7)	0
Acute respiratory distress syndrome^a,b^	1 (0.7)	0
Dyskinesia	0	2 (1.3)
Parkinson's disease	0	1 (0.6)
Back pain	0	1 (0.6)
Hepatic enzyme increased	1 (0.7)	0
Acute pancreatis^b^	1 (0.7)	0
Pneumonia^b^	1 (0.7)	0

a led to death, ^b^occurred in same patient.

This randomized study with long-term follow-up in early, stable PD suggests that OPC's levodopa dose-stabilizing effect persist for at least 76 weeks. While numerical advantages in motor scores and complications favored earlier OPC, the trial was not powered to compare early versus delayed initiation nor to assess delayed onset of motor complications, which would require larger samples and longer duration.^3,5^ Nonetheless, similar findings have been reported previously, such as a modest 1.3-point advantage in motor scores at 12 months with earlier entacapone treatment.^6^ Although numerical advantages for earlier OPC initiation in motor complications were observed, the study duration was insufficient for definitive conclusions. We cannot discount the possibility that the low level of motor complications observed in the short-medium term was due to slow disease progression.

Despite the fact that COMT inhibitors have been consistently efficacious in improving motor scores in early, stably treated patients,^1,7–9^ the shorter time to motor complications seen in the STRIDE-PD study^3^ led to the general dismissal of any advantage for early COMT inhibition in patients without motor complications. As previously reported, adjunct OPC improved motor scores by 6–7 points versus double-blind baseline,^1^ exceeding the proposed minimal clinically relevant difference (MCRD) of −3.25 points.^10^ We now show that this enhanced motor function is maintained over 76 weeks of continuous OPC treatment without increasing the risk of motor complications and is well tolerated.

## References

[1] Ferreira JJ Rascol O Stocchi F , et al. Opicapone as adjunct to levodopa in treated Parkinson's disease without motor complications: a randomized clinical trial. Eur J Neurol 2025; 32: e16420. 10.1111/ene.16420 PMC11718218 39790009

[2] Lees A Ferreira JJ Rocha JF , et al. Safety profile of opicapone in the management of Parkinson's disease. J Parkinsons Dis 2019; 9: 733–740. 31498127 10.3233/JPD-191593

[3] Stocchi F Rascol O Kieburtz K , et al. Initiating levodopa/carbidopa therapy with and without entacapone in early Parkinson disease: the STRIDE-PD study. Ann Neurol 2010; 68: 18–27. 20582993 10.1002/ana.22060

[4] Olanow CW Kieburtz K Rascol O , et al. Factors predictive of the development of levodopa-induced dyskinesia and wearing-off in Parkinson's disease. Mov Disord 2013; 28: 1064–1071. 23630119 10.1002/mds.25364

[5] Jenner P Rocha JF Ferreira JJ , et al. Redefining the strategy for the use of COMT inhibitors in Parkinson's disease: the role of opicapone. Expert Rev Neurother 2021; 21: 1019–1033. 34525893 10.1080/14737175.2021.1968298

[6] Nissinen H Kuoppamäki M Leinonen M , et al. Early versus delayed initiation of entacapone in levodopa-treated patients with Parkinson’s disease: a long-term, retrospective analysis. Eur J Neurol 2009; 16: 1305–1311. 19570145 10.1111/j.1468-1331.2009.02726.x

[7] Waters CH Kurth M Bailey P , et al. Tolcapone in stable Parkinson's disease: efficacy and safety of long-term treatment. The tolcapone stable study group. Neurology 1997; 49: 665–671. 9305320 10.1212/wnl.49.3.665

[8] Hauser RA Panisset M Abbruzzese G , et al. Double-blind trial of levodopa/carbidopa/entacapone versus levodopa/carbidopa in early Parkinson's disease. Mov Disord 2009; 24: 541–550. 19058133 10.1002/mds.22343

[9] Brooks DJ Sagar H . Entacapone is beneficial in both fluctuating and non-fluctuating patients with Parkinson's disease: a randomised, placebo controlled, double blind, six month study. J Neurol Neurosurg Psychiatry 2003; 74: 1071–1079. 12876237 10.1136/jnnp.74.8.1071 PMC1738605

[10] Horváth K Aschermann Z Ács P , et al. Minimal clinically important difference on the motor examination part of MDS-UPDRS. Parkinsonism Relat Disord 2015; 21: 1421–1426. 26578041 10.1016/j.parkreldis.2015.10.006

